# Self-reported musculoskeletal disorders and associated factors among HIV/AIDS patients following ART at University of Gondar Comprehensive Specialized Hospital, Gondar, Ethiopia, 2021: Aa cross-sectional study design

**DOI:** 10.1186/s12879-023-08497-1

**Published:** 2023-08-18

**Authors:** Alemu Kassaw Kibret, Melisew Mekie Yitayal, Getachew Azeze Eriku, Moges Gashaw, Ermias Solomon Yalew, Fkrte Kebede Weldetsadik

**Affiliations:** Department of Physiotherapy, School of Medicine, College of Medicine and Health Sciences, University of Gondar, Gondar, Ethiopia

**Keywords:** Musculoskeletal disorder, HIV/AIDS patients, Anti-retroviral medication, Ethiopia

## Abstract

**Background:**

Musculoskeletal disorders is an inflammatory, degenerative diseases and disorders that cause pain and functional impairments. Musculoskeletal disorders are common and the major global health concern among people with human immunodeficiency virus/acquired immunodeficiency syndrome which causes physical disability. Despite, it is a recognized health problem among human immunodeficiency virus-positive patients, there is a lack of data on musculoskeletal disorders among patients following anti-retroviral therapy in sub-Saharan Africa, particularly Ethiopia. Therefore, the main aim of the study was to assess the prevalence and associated factors of musculoskeletal disorders among adult human immunodeficiency virus-positive patients following anti-retroviral therapy.

**Method:**

An institutional-based cross-sectional study was conducted from September 1st to October 1st, 2021 at University of Gondar Comprehensive Specialized Hospital, Gondar, Ethiopia. The data was collected through an interview-administered questionnaire and patient medical record review of 324 participants. Binary logistic regression was used to identify associated risk factors of musculoskeletal disorders. The strength of the association was detected by the adjusted odds ratio and P-value.

**Result:**

The annual prevalence of musculoskeletal disorders among participants was 158 (48.5%) with [95% CI: 43%, 54%], opportunistic infection [AOR, 10.43; 95% CI = 2.76–42.25], type of ART medication used, CD4-count [AOR, 0.13; 95% CI 0.03–0.85], and change in anti-retroviral therapy regimen change [AOR, 8.14; 95%CI 2.06–32.09] were significantly associated with musculoskeletal disorders.

**Conclusion:**

The prevalence of musculoskeletal disorders was moderate. Recent CD4 count, opportunistic infection, antiretroviral therapy regime at initiation, and anti-retroviral therapy regime change were significantly associated with musculoskeletal disorder. A multidisciplinary approach is required for preventing and treating musculoskeletal disorders among human immunodeficiency virus-positive patients following anti-retroviral therapy.

## Background

Musculoskeletal disorders (MSDs) encompass a variety of conditions caused by inflammatory, degenerative, genetic and autoimmune diseases that affect the muscles, tendons, ligaments, joints, peripheral nerves and supporting blood vessels in the body [1, 2]. Musculoskeletal disorders are the most prevalent and major global health concern, causing physical impairment [3], as well as human misery that impacts the physical quality of life, social engagement, psychological well-being, and financial consequences [4, 5]. Furthermore, MSDs are the major cause of work impairment, absenteeism from work, and loss of performance in many jobs [6], resulting in significant economic consequences for the working-age population [7].

The introduction of ART (Anti-retroviral therapy) in the late 1990s substantially increased the average lifespan of persons living with HIV/AIDS (human immunodeficiency virus/acquired immunodeficiency syndrome). The majority of people who follow the recommended ART protocol achieve immunological improvement and have a normal life expectancy [8–11]. With this improved life expectancy has accompanied an increase in symptom load among persons living with HIV infection, such as musculoskeletal pain/disorder [12–15]. HIV/AIDS’ immunosuppressive properties and direct viral activities on specific body systems, combined with complicated environmental and genetic interactions, increase the vulnerability of HIV/AIDS patients to musculoskeletal disorders [16]. People living with HIV/AIDS may also experience pain as a result of the virus’s direct effects on the peripheral or central nerve systems, opportunistic infections, and ART adverse effects. The discomfort could also be idiopathic, with no evident cause, or caused by other disorders unrelated to HIV/AIDS [17, 18]. Many studies have found that musculoskeletal issues affect 72% of HIV/AIDS patients [19]. The prevalence of pain in AIDS patients in various hospital settings or in home care settings has been found to range from 54 to 83% [20].

The experience of living with HIV/AIDS also brings serious consequences to the patient’s quality of life and permeates all areas of life through anxiety, depression, stress, changes in sleep patterns, the breakdown of social and emotional relationships, and difficulties regarding sexuality. Because the environment can influence this dynamic, the experience of living with HIV/AIDS in places with different social, political, and economic conditions may also be factors that define the quality of life of these patients [21–23]. Different epidemiology studies revealed that, socio-demographic factor, (being older age, body mass index), CD4 count, having co-morbidity, opportunistic infection, duration of ART treatment, change ART regime had significant associated with musculoskeletal disorder among people living with HIV/AIDS [24–27].

Despite the high incidence of MSDs among HIV/AIDS patients following ART, there is paucity of data on the prevalence and associated factors of musculoskeletal disorder among HIV/AIDS following ART in sub-Saharan Africa particularly in the study area, Ethiopia. Finding from this study have a significant impact to design an appropriate treatment and prevention strategies to reduce the disabling effect of MSDs among people living with HIV/AIDS. Therefore, the aim of this study was to determine the prevalence and associated factors of MSDs among adult HIV/AIDS patients following ART clinic at University of Gondar comprehensive specialized hospital, Gondar, Ethiopia, 2021.

## Methods

### Study design and setting

An institutional-based cross-sectional study was conducted from September 1st to October 1st 2021 at University of Gondar comprehensive specialized hospital, Gondar, Ethiopia. Gondar city is found in Amhara regional state,727km away from Addis Ababa the capital city of Ethiopia. According to the 2015 census conducted by the central statistical agency of Ethiopia, Gondar had a total population of 206,987(29). University of Gondar comprehensive specialized hospital is the one and only teaching referral hospital in Gondar city, which provides service for more than 5 million people. The HIV care services of the hospital were established in 2003 and have three clinics that includes adult ART, pediatrics ART, and Volunteer Counseling and Testing (VCT). A total of 1950 adult HIV-positive patients were following ART clinic at University of Gondar Comprehensive Specialized Hospital per month.

### Source population

All HIV- positive adult patients who were following ART clinic at University of Gondar Comprehensive Specialized Hospital were study’s source population.

### Study population

The study population included all HIV- positive adult patients who were following ART clinic at University of Gondar Comprehensive Specialized Hospital during the data collection period.

#### Inclusion criteria

This study included all selected HIV- positive adult patients aged at least 18 years who attended adult ART clinic and who were able to give informed consent.

### Exclusion criteria

Adult HIV-positive patients who seriously ill and unconscious, women patients who were pregnant, and those who had trauma or surgery of the past 6 months prior to data collection at any part of their body were excluded from the study.

### Sample size determination

The sample size was determined using single population proportion formula with assumptions of 95% confidence interval, 5% margin of error, and by taking anticipated prevalence of MSDs 50% since there were no studies in Ethiopia and sub-Saharan Africa. The Z-value of 1.96 was used at 95% CI. (n: sample size, P: proportion, d: marginal error).

$$\frac{{{(Z}_{a/2})}^{2}*P(1-P)}{{d}^{2}}$$

$$\frac{{\left(1.96\right)}^{2}*0.5\left(0.5\right)}{{0.05}^{2}}$$

$${\rm{Nx}} = 384$$

Using correction formula for less than 10,000 population.

$$n=\frac{N*Nx}{Nx+(N-1)} = 321$$

Where N = source population, Nx = sample size for unlimited population and n = corrected sample size. Adding 10% nonresponse rate the final sample size for the first objective becomes 354.

An odds ratio for the relationship between alcohol intake, presence comorbidity condition, gender and pain among HIV-positive patients were taken from previous study [28] to determine sample size for the second objective which is determining the associated factors of MSDs among HIV/AIDS patients taking ART. Power calculations for sample size using EPI Info version 7 with a 95% level of significance and 80% power were used. The calculations produced sample sizes ranging from 80 to 139 for testing associations between the presence of pain and the variables of presence comorbidity, condition alcohol intake, and gender. By comparing sample size of the first and second objectives, sample size of the first objective were taken.

Therefore, the final sample size of the study was 354.

#### Sampling technique and procedure

Systematic random sampling was used to enumerate the study subjects, “K” was calculated to be 5, (k-value was determined by dividing the total number of adult HIV-infected patients to the actual sample size). Allocating randomly from 1 to 5 and by drawing with lottery method to determine the starting unit (3) then after arranging the patients based on the order of coming to the clinic, each study participant was selected every 5 intervals.

### Variables

#### Dependent variable

Musculoskeletal disorder (yes/no).

### Independent variables

#### Socio-demographic factors

age, sex, religion, marital status, educational level, salary and ethnicity.

#### Individual and lifestyle characteristics of participants

BMI, Physical exercise, alcohol drinking and cigarette smoking.

#### Clinical and treatment-related

Total duration since HIV/AIDS confirmed, baseline CD4 count, baseline WHO clinical stage, WHO clinical stage at ART initiation, recent WHO clinical stage, opportunistic infections, total time of using ART drug, and ART regime at initiation.

### Operational definition

#### Musculoskeletal disorder

if the patient experiences a complaint of unpleasant sensation(pain), ache, discomfort interfering with their activity of daily life at any part of their body region( neck, shoulder, elbow, wrist, back, hip, knee, ankle and foot) at any time during the last twelve months [29].

#### BMI

weight in kilogram divided by the square of the height in meters (kg/m2) underweight < 18.50, normal 18.50-24.99, overweight > = 25 [30].

#### Alcohol drinking

Someone taking any kind of alcohol more than about two bottles of beer at least twice per week [31].

#### Physical exercise

-Doing regular moderate to vigorous physical activity of 30 to 45 min duration 3 to 5 days per week[32].

#### Cigarette smoking

-Someone who smokes cigarettes in the last time and currently smokes more than two days or every day per week[33].

### Data collection instrument

Face to face interview was deployed to assess the patient’s background information, which consist socio-demographic and socioeconomic questions. Questionnaires were developed from different literatures. Patient’s clinical and laboratory information (which contains and of all HIV patients under ART follow-up including a detailed antiretroviral therapy history) was collected by reviewing of patient charts.

The study participant’s musculoskeletal disorder was assessed by using Nordic Musculoskeletal questioner to evaluate musculoskeletal symptoms. The questionnaire was designed to determine the prevalence of musculoskeletal issues in a certain population while also considering where they occur on the body. The questionnaire had three sections: Section I: - socio-demographic, section II: -personal and lifestyle characteristics, and section III: - clinical and treatment characteristics [29].

### Data processing and analysis

Descriptive statistics were obtained, and the results were provided in the form of frequency, percentage, mean, and table. The connection of independent variables with MSDs among adult HIV-positive patients was demonstrated using bivariate and multivariable logistic regression analysis. To identify the variables attributed with the outcome variable, associations among independent variables and dependent variables were initially examined using bivariate logistic regression analysis. The prospective candidate variables having a p-value of 0.2 in the bivariate analysis were then incorporated into the multivariable analysis. Finally, variables with *p* < 0.05 in the final logistic model were considered statistically significant and the strength and direction of association were measured by adjusted odds ratio (AOR) with corresponding 95% confidence interval. Model fitness was checked through Hosmer–Lemeshow goodness of fit test and it was fitted.

### Data quality control

The questionnaire was first prepared in English and translated into the local language Amharic and back translation to English to ensure conceptual integrity. Data were collected by four (BSc physiotherapists) and one supervisor (MSc physiotherapist). Both the data collectors and the supervisor were trained for one day on the objective, methodology, the importance of privacy, ensuring the confidentiality of the respondents and data collection approach of the research prior to the actual data collection. Before the data was officially collected, the questionnaire was pre-tested out of the study area on 5% (18) of the total sample size among the ART users in Bahir Dar city at Felege Hiwet referral hospital. Discussion was held and changes made based on the findings of the pre-test like making questions clear to the patients. The supervisors made routine checkups for completeness and consistency of the data. The questionnaire was reviewed and checked for completeness, accuracy and consistency by the supervisors and investigators to take timely corrective measures.

### Result

#### Socio-demographic characteristics of study participants

Three hundred fifty-four HIV/AIDS patients at the ART clinic were selected for the study yielding a response rate of 324 (91.5%). Among the participants, the majority were female 208(64.2%), in the age range of 25–44 years (48.5%), and most were married 290 (58.6%)). The income statuses of the participants were mean of 2623 birr (SD ± 0.87) and the majority had an income > 2000 birr (39.8%), completed secondary school 96(29.6%)), and regarding religion 259(79.9%)) were Orthodox. See (Table 1).

**Table 1:** Socio-demographic characteristics of study participants (n = 324)

Variables	Frequency(n)	Percent (%)
**Age**		
< 25 25–44 > 44	27 157 140	8.3 48.5 43.2
**Sex**		
Male Female	116 208	35.8 64.2
**Religion**		
Orthodox Muslim Others	259 50 15	80.0 15.4 4.6
**Marital status**		
Single Married Divorced Widowed	79 190 32 23	24.4 58.6 9.9 7.1
**Educational level**		
Unable to read and write Primary school Secondary school Tertiary school and above	65 90 96 73	20.1 27.8 29.6 22.5
**Monthly Income (Ethiopian Birr)**		
< 1000 1000–2000 > 2000	114 81 129	35.2 25.0 39.8

### Individual and lifestyle characteristics of participants

Among study respondents, the majority 320 (98.8%) were nonsmokers and 311 (96%) were nonalcoholic. Regarding physical exercise, the majority 237(73.1%) were not doing physical exercise and most of them had a normal BMI of 212(65.4%). See (Table 2).

**Table 2:** Personal and lifestyle characteristics of study participants (n = 324)

Variable	Frequency (n)	Percent (%)
**BMI**		
Underweight Normal Overweight Obese	62 212 40 10	19.2 65.4 12.3 3.1
**Physical exercise**		
Yes No	87 237	26.9 73.1
**Cigarette smoking**		
Yes No	4 320	1.2 98.8
**Alcohol use**		
Yes No	13 311	4.0 96.0

### Clinical and treatment-related characteristics of study participants

About 65.1% of the participants had HIV/AIDS for about greater than 9 years. The majority of the participant had baseline CD4 cell count 200–350(47.2%), CD4 count at ART initiation 200–350(47.5) and the recent CD4 count was 611 cell/mm3. About 43(13.3%) and 25(7.7%) % had chronic comorbidities and opportunistic infection respectively. The majority of the participants were less than 10 years (53.1%) since using ART drugs. AZT-3TC-NVP (1c) is the most commonly used ART regimen at initiation (113(34.9%)), and the majority of the participants had regime change 305(94.2%) and (315(97.2%) use first-line regimen currently. Most participants 218(67.3%) use prophylaxis and within these prophylaxis users, INH is commonly used (134(41.4%)). (See Table 3)

**Table 3:** Clinical and treatment-related characteristics of study participants (n = 324)

Variables	Frequency (n)	Percent (%)
**Total duration since confirmed**		
< 5 years 5-9 Year > 9 year	54 59 211	16.7 18.2 65.1
**Baseline CD4 count**		
< 200 cell/mm3 200–350 cell/mm3 > 350 cell/mm3	69 153 102	21.3 47.2 31.5
**CD4 count at initiation**		
< 200 cell/mm3 200–350 cell/mm3 > 350 cell/mm3	66 154 104	20.4 47.5 32.1
**Resent CD4 count**		
< 200 cell/mm3 200–350 cell/mm3 > 350 cell/mm3	4 18 302	1.2 5.6 93.2
**Baseline WHO clinical stage**		
Stage l Stage ll Stage lll Stage lv	173 66 71 14	53.4 20.4 21.9 4.3
**WHO clinical stage at ART initiation**		
Stage I Stage II Stage III Stage IV	178 65 67 14	54.9 20.1 20.7 4.3
**Recent WHO clinical stage**		
Stage I Stage II Stage III	314 6 4	96.9 1.9 1.2
**Opportunistic infection**		
Yes No	25 299	7.7 92.3
**Total time of using ART drug**		
< 10 10–20 > 20	172 143 9	53.1 44.1 2.8
**ART regime at initiation**		
D4t-3TC-NVP (1a) D4T-3TC-EFV (1b) AZT-3TC-NVP (1c) AZT-3TC-EFV(1d) TDF-3TC-EFV (1e) TDF-3TC-NVP (1f)	37 28 113 66 57 23	11.4 8.6 34.9 20.4 17.6 7.1
**Any art regime change**		
Yes No	305 19	94.1 5.9
**Current ART regime you use**		
First line Second line	315 9	97.2 2.8
**Use of prophylaxis**		
Yes No	218 106	67.3 32.7
**Type of prophylaxis**		
INH CPT	134 84	41.4 25.9

### Prevalence of MSD among HIV/AIDS patients following ART

**Fig. 1:**
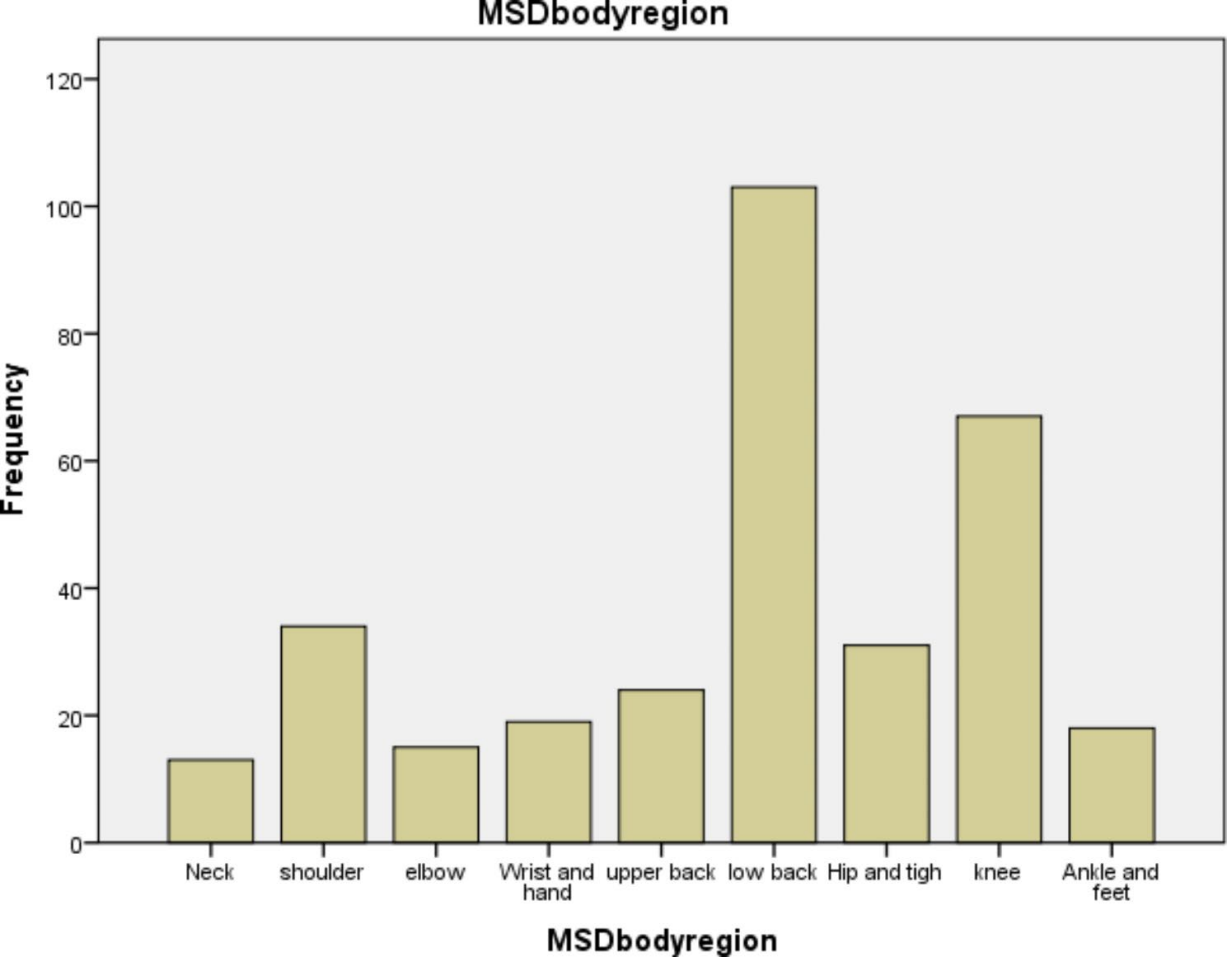
Prevalence of MSDs distribution among different body regions of HIV/AIDS patients following ART (n = 324)

Overall, the annual prevalence rate of musculoskeletal disorder among HIV/AIDS patients following ART at University of Gondar comprehensive specialized hospital was found 48.5%. The annual prevalence of MSD distribution among their body parts was lower back (31.8%), knee (20.7%), shoulder (10.6%), hip and thigh 9.5%, ankle and foot 8.9%, upper back 7.4%, hand and wrist 5.9%, elbow 4.5%, and neck 4%. (See Fig. 1)

### Bivariate and multivariable logistic regression analysis of associated factors of MSD among HIV/AIDS patient following ART

First, all variables were taken in to bivariate logistic regression analyses. Of all variables eleven variables were significantly associated with MSD of HIV/AIDS patients following ART clinic: marital status, educational level, total duration since HIV confirmed, baseline CD4 count, most recent CD4 count, baseline WHO clinical stage, WHO clinical stage at ART initiation, chronic co-morbidities, opportunistic infection, ART regime at initiation and ART regime change were significant to the experience of musculoskeletal disorder and among those significant variables four of them: recent CD4 count, opportunistic infection, ART regime at initiation and ART regime change were clinically significant in multi variable analysis at 95% confidence interval. See (Table 4).

**Table 4:** Results of bivariate and multivariable logistic regression analysis of associated factors of MSDs among HIV/AIDS patients following ART (n = 324)

Variables	Musculoskeletal disorders		COR (95% CI)	AOR (95% CI)	P-value
	Yes (n)	No (n)			
**Age**					
< 25 25–44 > 44	12 90 65	15 67 75	1 1.68 (0.74–3.82) * 1.08 (0.47–2.48)	1 1.81 (0.55–5.97) 0.97 (0.55–1.72)	1 0.330 0.927
**Sex**					
Male Female	54 103	62 105	1 1.13 (0.72–1.78)		
**Religion**					
Orthodox Muslim Others	126 25 6	133 25 9	0. 14 (0.52–1.74) 1.42 (0.56–5.23) 1		
**Marital status**					
Single Married Divorce Widow	41 103 17 17	38 87 15 6	1 1.10 (1.09–8.56) * 1.50 (1.27–8.89) * 2.62 (1.01–10.23) *	1 0.37 (0.10–1.35) 0.36 (0.11–1.17) 0.34 (0.09–1.36)	1 0.132 0.088 0.128
**Education**					
Primary Secondary Tertiary Unable to read and write	50 47 41 29	40 49 32 36	1 0.77 (0.43–1.37) 1.03 (0.55–1.91) 0.64 (0.34–1.23) *	1 0.59 (0.28–1.28) 0.74 (0.34–1.64) 0.44 (0.19–1.01)	1 0.184 0.460 0.0551
**Salary in Ethiopian birr**					
< 1000 1000–2000 >2000	63 42 62	51 39 67	1.34 (0.81–2.21) 1.16 (0.67–2.03) 1		
**Physical Exercise**					
Yes No	45 112	42 125	1 0.84 (0.51–1.37)		
**BMI**					
Underweight (< 18.50) Normal (18.50-24.99) Overweight (25 -29.9) Obese (≥ 30)	36 111 12 8	26 101 28 2	0.35 (0.07–1.77) 0.28 (0.06–1.32) 0.11 (0.02–1.58) 1		
**Use Alcohol**					
Yes No	6 161	7 150	0.79 (0.26–2.43) 1		
**Duration since confirmed**					
< 5year 5–9 Year > 9 year	20 29 108	34 30 103	1 1.64 (0.96–3.29) * 1.78 (0.61–1.93)	1 0.94 (0.44–1.99) 1.03 (0.51–2.05)	1 0.880 0.940
**CD4 Count at ART Initiation**					
< 200 cell/mm3 200–350 cell/mm3 > 350 cell/mm3	31 78 58	35 76 46	0.702(0.38–1.30) 0.814(0.49–1.34) 1		
**The Most Recent CD4**					
< 200 cell/mm3 200–350 cell/mm3 > 350 cell/mm3	2 14 151	2 4 151	1.00 (0.14–7.19) 3.50 (1.13–10.88) * 1	0.54 (0.04–6.83) 0.13 (0.03–0.85) 1	0.637 0.004** 1
**Baseline WHO Clinical Stage**					
Stage I Stage II Stage III Stage IV	96 35 28 8	77 31 43 6	1 0.91(0.51–1.59) 0.52 (0.29–0.92) * 1.07(0.36–3.21)	1 0.33 (0.01–7.65) 0.86 (0.04–18.46) 1.69 (0.43–6.71)	1 0.490 0.920 0.460
**WHO Clinical Stage at ART Initiation**					
Stage I Stage II Stage III Stage IV	97 35 27 8	81 30 40 6	1 0.99 (0.551–1.72) 0.56 (0.32–0.99) * 1.11 (0.37–3.34)	1 3.51(0.20-60.56) 0.99 (0.06–15.59)	1 0.387 0.990
**Opportunistic infection**					
Yes No	4 163	21 136	0.16 (0.05–0.47) * 1	10.43(2.76–42.25) 1	0.001** 1
**Duration of ART use**					
< 10 10–20 > 20			1.62 (0.42–6.23) 1.07 (0.28–4.16) 1		
**ART Regimen at Initiation**					
D4t-3TC-NVP (1a) D4T-3TC-EFV (1b) AZT-3TC-NVP (1c) AZT-3TC-EFV(1d) TDF-3TC-EFV (1e) TDF-3TC-NVP (1f)	15 12 48 38 28 16	22 16 65 28 29 7	1 1.1 3.35 (1.10-10.12) * 1.08 3.05 (0.95–9.74) * 1.99 3.09 (1.18–8.11) * 1.42 1.68 (0.61–4.64) 3.35 2.37 (0.84–6.62) *	1 0.188(0.05–0.73) 0.189(0.04–0.76) 0.22(0.06–0.72) 0.43(0.12–1.51) 0.43(0.12–1.52)	1 0.016** 0.019** 0.012** 0.186 0.190
**ART Regimen Change**					
Yes No	152 15	153 4	1 3.77 (1.22–11.63) *	1 8.14 (2.06–32.09)	1 0.003**
**Current ART Regimen**					
First line Second line	161 6	154 3	1 1.91 (0.47–7.78)		
**Prophylaxis (INH & CPT) used**					
Yes No	110 57	118 49	1 1.25 (0.55–1.39)		

HIV/AIDS patients with opportunistic infections had 10 times higher odds of MSDs than patients without opportunistic infection (AOR = 10.43, 95% CI = 2.76 to 42.25). In addition, compared to those who used D4t-3TC-NVP (1a) type of ART, patients who was taking D4T-3TC-EFV (1b) was associated with an 81% reduction in the odds of developing MSDs [AOR = 0.19, 95% CI = 0.05 to 0.73], AZT-3TC-NVP (1c) had similarly an 81% reduction in the odds of developing MSDs [AOR = 0.189, 95% CI = 0.047 to 0.76] and AZT-3TC-EFV(1d) had 78% reduction in the odds of developing MSDs [AOR = 0.22, 95% CI = 0.067to 0.72].

Furthermore, Those HIV patients who didn’t change their first ART treatment regime had 8 times higher odds of developing musculoskeletal disorders compared to those who changed their first treatment regime [AOR = 8.14, 95% CI = 2.062 to 32.09. Patient with recent CD4 count 200–350 cells/mm3 had an 87% reduction in the odds of developing musculoskeletal disorders than patients with recent CD4 count > 350 cell/mm3. (See Table 4)

## Discussion

The study examines the annual prevalence and associated factors of musculoskeletal disorders among HIV/AIDS patients following ART. According to our study the annual prevalence of musculoskeletal disorders among HIV/AIDS patients following ART at university of Gondar specialized compressive hospital in Gondar city, Ethiopia was 48.5% with [95% CI: 43%, 54%]. Opportunistic infection, type of ART medication used, CD4-count, and change in ART regimen change is significantly associated with musculoskeletal disorder among HIV/AIDS patients following ART.

Comparisons between different studies in literature are difficult for several reasons: differences in description of pain localizations, differences in pain definitions, different selection of samples, and varied demographic differences across groups. Although these differences influence results, the overall prevalence is lower than the finding conducted in Denmark, India and Zambia [15, 34, 35]. This could be due to the evidence in Denmark used longitudinal study design that avoids recall bias and includes pain reports from headache and gastro-intestinal body region and most study participants in Indian study were truck drivers that leads to work related musculoskeletal disorder [36] which rises the occurrence of musculoskeletal disorder among HIV/AIDS patients. In addition, our study finding is lower than the study in Zambia (61%) since our study didn’t consider stiffness and fatigue as disorder which makes their finding higher. Our study is also lower than finding from systematic review [20]. The possible explanation might be our study investigated the annual prevalence which might lead for higher recall bias though the systematic review includes studies done on three months recall period.

The current study showed that opportunistic infection as predictor of musculoskeletal disorders where those who had opportunistic infection had higher odds of MSDs among HIV/AIDS patients following ART than those who didn’t have. This could be due to opportunistic infection may leads to the decrease in immune system which will finally predisposes for the occurrence of musculoskeletal disorders [37], and those with opportunistic infection also be inactive that leads for joint pain, stiffness, discomfort, and poor self-care which finally result in musculoskeletal disorders.

In addition, ART regimen used like D4T-3TC-EFV (1b) (Stavudine, lamivudine, and efavirenzes), AZT-3TC-NVP (1c) (zidovudine, lamivudine, and nevirapine), and AZT-3TC-EFV(1d) (zidovudine, lamivudine efavirenzes) were significantly associated with musculoskeletal disorders compared to D4t-3TC-NVP (1a) (Stavudine, lamivudine, and Nevirapine). This might be due to higher adverse effect of D4t-3TC-NVP (1a) like toxicity that leads to peripheral neuropathy which is characterized by pain and discomfort in any parts of their body [38] .

Furthermore, HIV/AIDS patients who didn’t change their first ART regimen had higher odds of developing musculoskeletal disorders than those who changed their first ART regime. If ART initiated and remains in medication indefinitely there might be acute and chronic toxicities, concomitant clinical conditions, and development of virologic failure [39]. Our study revealed CD-4 count between 200 and 350 were significantly associated with musculoskeletal disorder compared with those who had CD-4 count > 350. The current study finding is in contrary of previous evidences where a decrease in CD4 count means the body experiences decreased immunity which may lead to musculoskeletal infections and disorders [40]. The possible explanation might be due to small sample size the current study.

## Conclusion

The prevalence of musculoskeletal disorder was moderate and the following factors are clinically significantly associated with musculoskeletal disorders among ART patient who are following at ART clinic: Recent CD4 count, opportunistic infection, ART regime at initiation and ART regime change. Thus HIV/AIDS patients who follow ART should be advised and educated to prevent opportunistic infection, use ART regime that have less impact in MSDs and to change their ART regime to prevent the long-term toxic effect of the drug. In addition, we recommend researchers to further investigate the effect of CD4 count for MSDs among HIV/AIDS patients following ART.

### Strength and limitation of the study

This study assessed the burden of musculoskeletal disorder among HIV/AIDS patients following ART, which is the first study in Ethiopia. However, this study has certain limitations, the main limitation of this study was including small sample size. The cross-sectional design of the study precludes a follow-up, which would have provided a better design for detecting characteristics associated with musculoskeletal disorders. The results were also based on self-reported data from patients. This could have been influenced by recall bias.

## Data Availability

The manuscript contains all of the data that is crucial to our findings. Requests for additional information on the dataset and questions about data sharing will be treated in accordance with a reasonable request to alemuphysio@gmail.com.

## List of Abbreviations

AIDS: Acquired immunodeficiency syndrome
AOR: Adjusted odds ratio
ART: anti-retroviral therapy
BMI: Body mass index
BSc: Bachelors of science
CI: Confidence interval
COD: Crude odds ratio
GBD: Global Burden of Disease
HIV: Human immunodeficiency Virus
MSc: Masters of science
MSD: Musculoskeletal disorder
MSK: Musculoskeletal
PLWHA: People living with HIV/AIDS
SPSS: Statistical Package for Social Sciences
UK: United Kingdom
VCT: Volunteer consulting and testing
WHO: World Health Organization
